# Supplementary material for: Autophagy Inhibition Induces the Secretion of Macrophage Migration Inhibitory Factor (MIF) with Autocrine and Paracrine Effects on the Promotion of Malignancy in Breast Cancer
Source: Biology (Basel). 2020 Jan 18;9(1):20. doi: 10.3390/biology9010020 (PMC7169388; doi:10.3390/biology9010020)
Supplement: Supplementary file 1 [file biology-09-00020-s001.zip › SupplementaryFigures.pdf]

Supplementary Figure 1

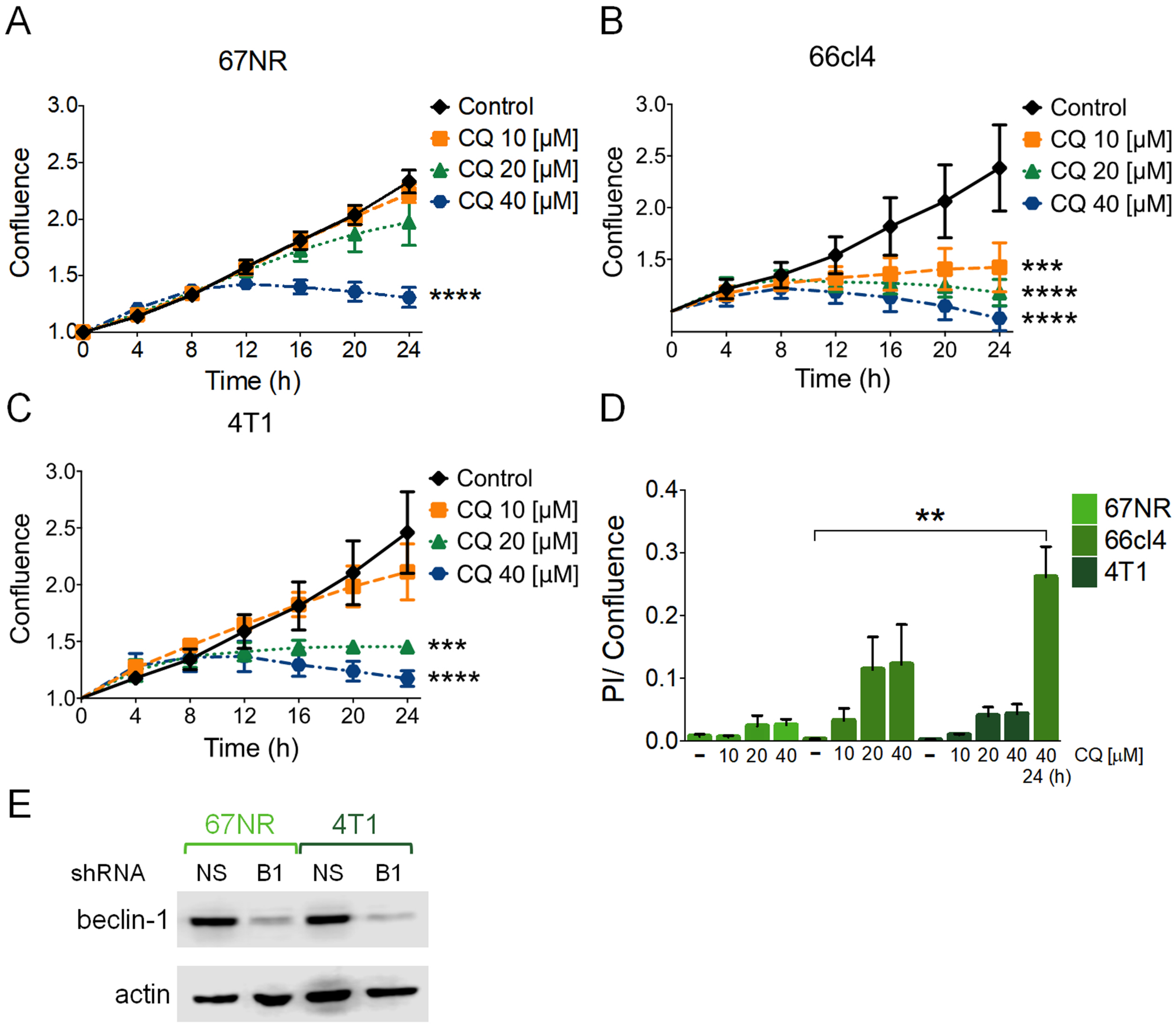

Supplementary figure 1. Proliferation curves of 67NR (**A**), 66cl4 (**B**) and 4T1 (**C**) cell lines with chloroquine (CQ) treatment. Cell death was evaluated as percent confluency of propidium iodide (PI) positive cells normalized to total cell confluency at 16 h of treatment with CQ at the indicated concentrations. A positive control for cell death was included as 66cl4 cells were treated with 40 µM CQ at 24 h (**D**). Knockdown efficiency was evaluated with a Western Blot for beclin-1 protein levels in the 67NR and 4T1 cell lines (**E**). Graphs show mean +/- standard error of four independent experiments. ***p<0.001, ****p<0.0001.

Supplementary Figure 2

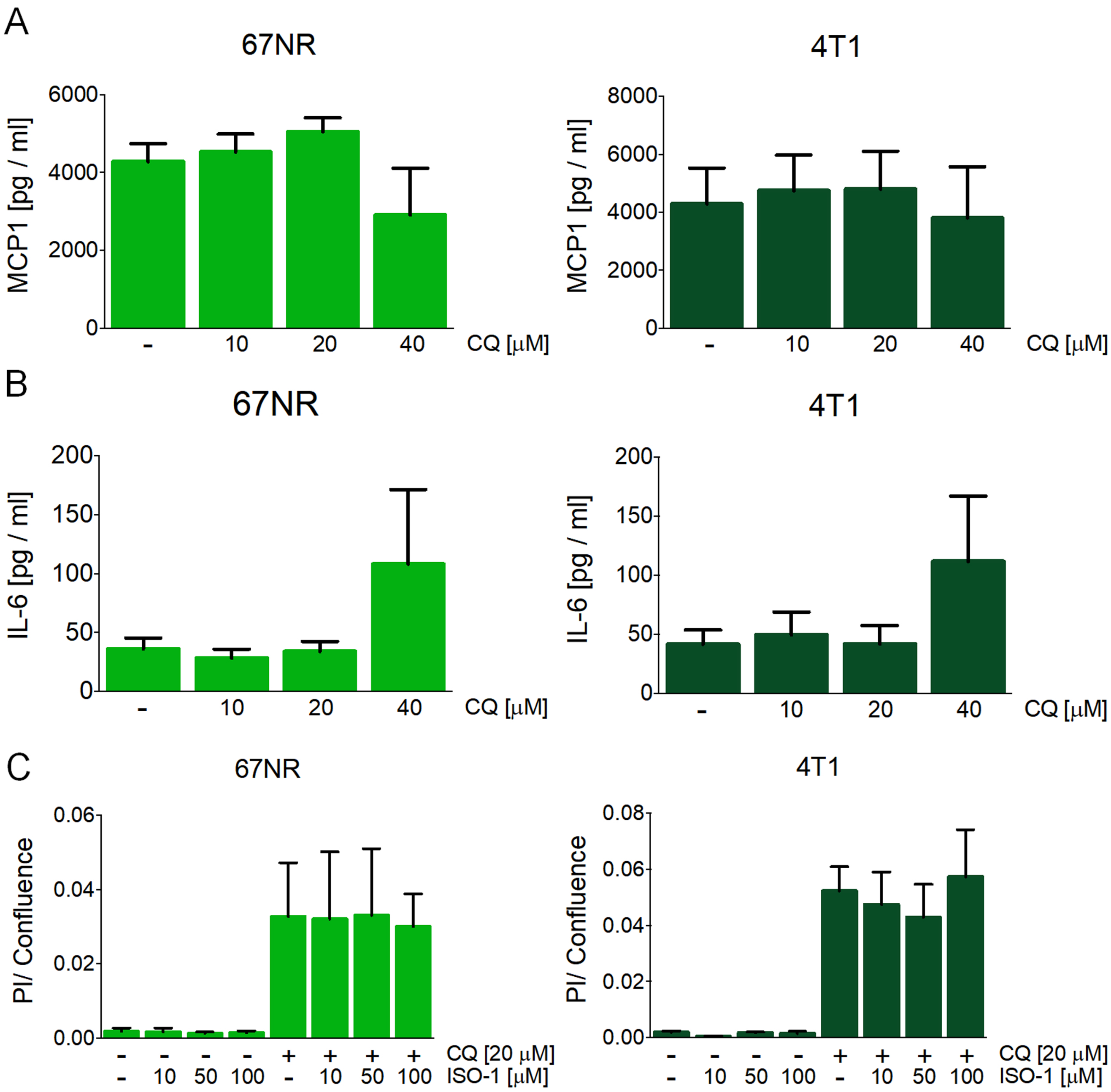

Supplementary figure 2. Secretion of MCP1 or IL-6 (A,B) in 67NR and 4T1 cell lines was not induced by CQ treatment. Secretion was evaluated in all cell lines after 16 h of treatment with CQ at the indicated concentrations using a human pro-inflammatory cytokine CBA kit by flow cytometry. Graphs show mean +/- standard error of three independent experiments. Cell death evaluation in 67NR or 4T1 (C) cells using 20 μM CQ and the indicated concentrations of ISO-1 after 24 h of treatment. Graphs show mean +/- standard error of three independent experiments; *p<0.05, **p<0.01.

Supplementary Figure 3

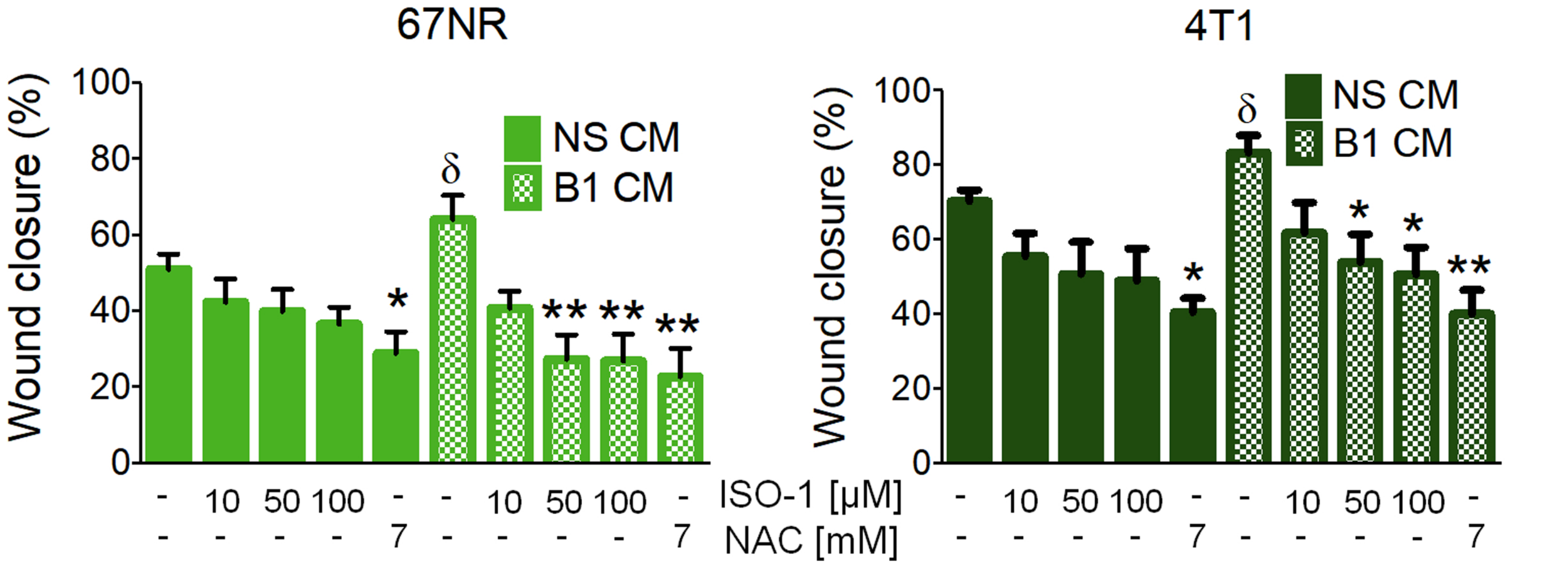

Supplementary figure 3. MIF secretion induced by the inhibition of autophagy with beclin-1 shRNAs paracrinally induced cell migration in autophagy proficient breast cancer cell lines. Conditional medium (CM) collected for 16 h from 66cl4 cells transduced with a non-silencing (NS CM) or beclin-1 (B1 CM) shRNA, was added to wild type 67NR or 4T1 cells for migration evaluation in a scratch-wound assay. Increased migration induced by B1 CM from 66cl4 cells was decreased in 67NR or 4T1 cells when 66cl4 beclin-1 shRNA expressing cells were also treated with an antioxidant (NAC) or when a MIF inhibitor (ISO-1) was added to the CM at the indicated concentrations. Graphs show wound closure area covered by the migrating cells at the indicated times or at 24 h. Graphs show mean +/- standard error of three independent experiments, *$p<0.05$, **$p<0.01$, δ: different from NS CM untreated control, *: different from their respective untreated control.

Supplementary Figure 4

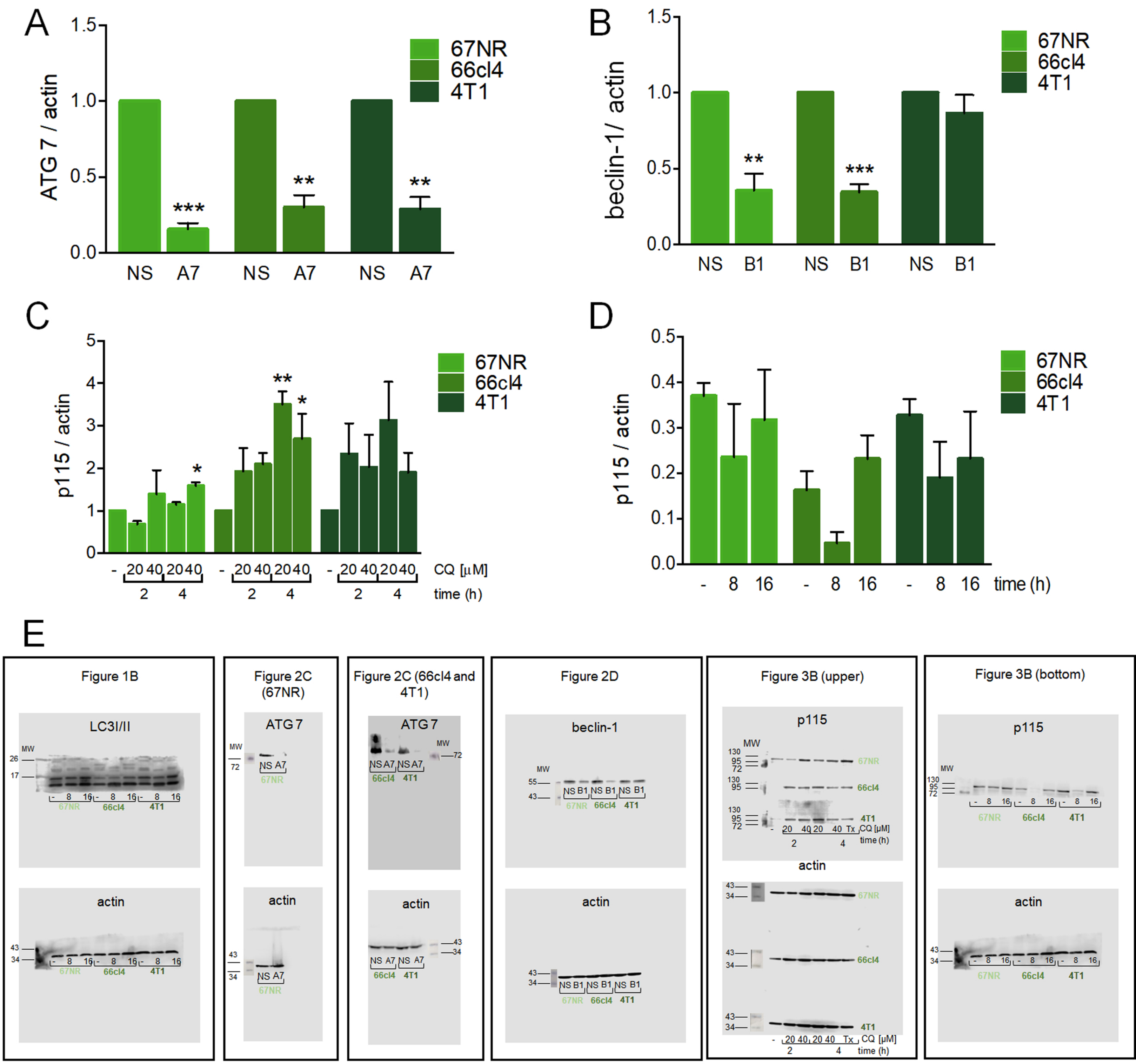

Supplementary figure 4. Densitometric analysis and complete membranes for all the Western Blots included in the manuscript. In A, B and C, the graph shows mean +/- standard error of three independent experiments. In D, the graph shows mean of two independent experiments. In E, the complete Licor image is shown without brightness or contrast changes for all Western Blots shown in the manuscript. Where more than one set of bands in shown (p115), three separate menbranes were cut at the area corresponding to the expected protein weight and the three membranes were exposed and imaged together. *p<0.05, **p<0.01, ***p<0.001. NS, non-silencing shRNA; A7, ATG 7; B1, beclin-1.
